# TiO_2_ and PEEK Reinforced 3D Printing PMMA Composite Resin for Dental Denture Base Applications

**DOI:** 10.3390/nano9071049

**Published:** 2019-07-22

**Authors:** Sheng-Gui Chen, Junzhong Yang, Yong-Guang Jia, Bingheng Lu, Li Ren

**Affiliations:** 1 School of Materials Science and Engineering, South China University of Technology, Guangzhou 510641, China; 2 National Engineering Research Center for Tissue Restoration and Reconstruction, Guangzhou 510006, China; 3 School of Mechanical Engineering, Dongguan University of Technology, Dongguan 523808, China

**Keywords:** 3D printing, PMMA composite resin, titanium dioxide, PEEK, biocompatibility, antibacterial activity, blood compatibility

## Abstract

The future of manufacturing applications in three-dimensional (3D) printing depends on the improvement and the development of materials suitable for 3D printing technology. This study aims to develop an applicable and convenient protocol for light-curing resin used in 3D industry, which could enhance antibacterial and mechanical properties of polymethyl methacrylate (PMMA) resin through the combination of nano-fillers of surface modified titanium dioxide (TiO_2_) and micro-fillers of polyetheretherketone (PEEK). PMMA-based composite resins with various additions of TiO_2_ and PEEK were prepared and submitted to characterizations including mechanical properties, distribution of the fillers (TiO_2_ or/and PEEK) on the fractured surface, cytotoxicity, antibacterial activity, and blood compatibility assessment. These results indicated that the reinforced composite resins of PMMA (TiO_2_-1%-PEEK-1%) possessed the most optimized properties compared to the other groups. In addition, we found the addition of 1% of TiO_2_ would be an effective amount to enhance both mechanical and antibacterial properties for PMMA composite resin. Furthermore, the model printed by PMMA (TiO_2_-1%-PEEK-1%) composite resin showed a smooth surface and a precise resolution, indicating this functional dental restoration material would be a suitable light-curing resin in 3D industry.

## 1. Introduction

Digital dental medical technology is becoming the mainstream of dental prosthetics worldwide [1]. Digital three-dimensional (3D) printing technology has greatly improved the diagnostic rate and has shown great potential in personalized medicine [1,2]. Compared to traditional manufacturing, 3D printed prostheses have shorter production cycles and higher precision, maximizing the comfort of patients with dentures. Nowadays, light-curing technology accounts for approximately 75% of 3D printing dental applications, and light-curing resins are commonly used fillers and restorative materials in stomatology [3,4]. Polymethyl methacrylate (PMMA) is a commercial light-curing resins used in 3D printing industry due to its low odor, low irritancy, good flexibility, and low cost [5]. However, the inherent limitations of PMMA, such as large shrinkage rate during light-curing, large brittleness, poor mechanical properties, and low antibacterial activity, etc., have hindered its extensive clinical application [6,7,8].

Several studies have shown that the modification of the inorganic fillers can remarkably improve the bonding force between the fillers and the resins, thereby effectively enhancing the macroscopic properties of resins [8,9,10]. For example, hydroxyapatite (HA) whisker could improve the bonding with resin matrix through crack deflection, crack bridging, pinning, and whisker pull, thus enhancing the mechanical properties of the composite resin [11]. Silicon dioxide (SiO_2_) nano-fibers and micro-particles were able to form an overlapped network in bis-GMA/TEGDMA dental resins, effectively enhancing the mechanical strength and the wear resistance of the composite resin [12]. Polyetheretherketone (PEEK) is a semi-crystalline engineering plastic with excellent mechanical and thermal properties. PEEK has various advantages, such as lightweight, non-toxicity, corrosion resistance, low modulus close to natural bone, etc. It has been widely used as promising clinical implants in orthopedic applications [13,14,15,16,17,18,19,20,21,22]. Ouyang et al. [19] used concentrated sulfuric acid to fabricate a three-dimensional network on the PEEK surface and investigated the osseointegration and the antibacterial ability of sulfonation modified PEEK composite materials. This study showed that the PEEK composite containing small amounts of sulfur concentrations would promote proliferation and osteogenic differentiation of rat bone mesenchymal stem cells (rBMSCs). Another study showed that, with an addition of titanium dioxide, the marten hardness parameter of PEEK was remarkably enhanced, though the Marten hardness parameter of PEEK was still not comparable to the natural tooth structure [20]. Schwitalla et al. [21,22] further demonstrated that the prepared PEEK specimens reinforced with different fiber fillers were able to withstand axial pressure caused by the mastication process. Their results suggested that PEEK compounds would be promising restorative materials in dental implant applications. In addition, a recent study showed that the mechanical properties of PEEK were not affected by artificial saliva solution with different pH values over 30 days at 25 °C [23]. To the best of our knowledge, though there are some studies that suggested PEEK as a potential restorative material in the oral cavity [20,21,22,23,24,25], the introduction of PEEK into PMMA matrix in light-curing printing system has not been reported yet.

Due to the long-term synergistic effects of acid-producing bacteria and fermentable carbohydrates in the oral environment, the antimicrobial activity of the composite resin is highly desirable in clinical applications. Studies have shown that the incorporation of nano-antibacterial agents would be an effective approach to enhance the antibacterial activity of the restorative materials [8,9,26,27,28]. Titanium dioxide (TiO_2_) has also shown good antibacterial activity by incorporation into resins as the nano-fillers [8,29]. Nano-TiO_2_ is a semi-permanent antibacterial agent with good photocatalytic performance, which could generate active oxygen species to kill bacteria under ultraviolet light [30,31]. Other antibacterial agents, e.g., silver nanoparticles (NPs), have shown to gradually reduce the antibacterial effects as the agent dissolution [9,32,33]. Furthermore, several studies suggested the addition of TiO_2_ would improve the mechanical and the thermal performances of composite resins [34,35,36]. Therefore, nano-TiO_2_ might be an ideal additive to enhance the general antibacterial performance of the PMMA matrix resin.

In general, within a certain range of nano-fillers, the higher the content of nano-fillers is, the better the mechanical properties of the composite resin and the wear resistance will be. However, the aggregation and the dispersion of NPs in the processing and the transportation are one of the problems that limit their extensive applications, which can be solved by the surface modification of NPs [9,37,38,39]. A silane coupling agent is a class of low molecular weight organosilicon surface treatment agents that can interact with hydroxyl groups in inorganic materials and long molecular chains in organic polymers [38]. Therefore, by using a silane coupling agent, a “molecular bridge” can be built between the interface of the inorganic substance and the organic substance, thereby increasing the bonding strength of the composite materials [39]. In addition, a nano-filler modified with the silane coupling agent could improve the dispersibility of the fillers in a polymer, thereby reinforcing the mechanical properties of the composite resin.

In this study, we prepared reinforced PMMA composite resins with good antibacterial activity and enhanced mechanical performance; these composite resins would be easy to manufacture with a reasonable cost in 3D printing industry. The reinforced antibacterial and mechanical properties of PMMA resin base could be achieved by the combination of KH570 modified TiO_2_ nano-fillers and PEEK micro-fillers. In order to optimize the ratio of TiO_2_ and PEEK added into the PMMA resin, we prepared samples from Group-1 to Group-5 (as shown in Table 1). The results of these samples regarding their mechanical properties test, fracture cross-section analysis, antibacterial properties, cell compatibility, and blood compatibility were analyzed and discussed. In addition, a 3D printing test was performed using the PMMA composite resin with the best performance, and the printing outcome was compared to that of pure PMMA resin.

## 2. Materials and Methods

### 2.1. Materials

Dental resins of polymethyl methacrylate (PMMA), product code NDBAPI01000, were purchased from NextDent (Netherland) and used directly. Tetrabutyl titanate (C_16_H_36_O_4_Ti, ≥ 99%) was purchased from Shanghai Sinopharm Reagent Co., Ltd., Shanghai, China. PEEK powder (biomedical grade) was purchased from Shengda Plastic Raw Materials Trading Company (manufactured by ICI, London, UK). KH570 (CH_2_ = C(CH_3_)COOC_3_H_6_Si(OCH_3_)_3_, silane coupling agent, ≥ 97%) was purchased from McLean (Shanghai, China). Other lab chemicals and reagents such as hydrochloric acid (HCl, 36.5 wt. %), sodium hydroxide (NaOH, ≥ 99%), ethanol (C_2_H_5_OH, ≥ 99%), etc., were purchased from Sinopharm Group (Beijing, China).

### 2.2. Preparation of TiO_2_ and TiO_2_-KH570 NPs

The nano-TiO_2_ was prepared by the hydrothermal method as previously described [40]. Briefly, 34 mL of tetrabutyl titanate was slowly added to 100 mL of 4 mol/L HCl solution and stirred for 2 h. The suspension was clarified into two layers, and then the lower layer liquid was transferred into a 100 mL hydrothermal reaction kettle. The hydrothermal process reacted at 180 °C for 12 h. After the reaction was naturally cooled, the pH of the solution was adjusted to 7.4 using 1 M NaOH. The solution was then centrifuged, and the precipitates were washed twice with deionized water and ethanol, respectively. Finally, the white precipitate of nano-TiO_2_ was obtained by vacuum drying.

Then, 10.0 g of nano-TiO_2_ powder was poured into a round bottom flask with 300 mL of ethanol. The solution was sonicated for 5 min, and then 2.0 g of KH570 was added. The flask was heated to 80 °C, refluxed for 5 h, centrifuged, washed, and vacuum dried to obtain KH570 modified TiO_2_, labeled TiO_2_-KH570.

### 2.3. Characterizations of TiO_2_, TiO_2_-KH570, and PEEK

The crystal structures of nano-TiO_2_ and TiO_2_-KH570 were analyzed by XRD (D8 ADVANCE mode, Bruker, Karlsruhe, Germany) using the Cu Kα radiation (40 kV, 200 mA) with a scanning speed of 10°/min, and the angle (2θ) range was set to 5–90°. The changes of the functional group for nano-TiO_2_ and TiO_2_-KH570 were analyzed by FTIR (VERTEX70 mode, Bruker, Karlsruhe, Germany). The samples of TiO_2_ and TiO_2_-KH570 were mixed with potassium bromide (KBr) and ground to fine tablets. Each sample was scanned 10 times with a wavelength range of 500–4000 cm^−1^ and a resolution of 4 cm^−1^. The hydrophilic analyses of samples nano-TiO_2_, TiO_2_-KH570, and PEEK were measured by a contact angle measuring instrument (JC200C1 Powereach mode, Shanghai Zhongchen, Shanghai, China). The samples were dispersed in ethanol at a final concentration of 5 wt. % and ultrasonic dispersion for 10 min. Then, the film of each sample was cast on a glass slide and dried in a blast oven at 50 °C for 48 h. A contact angle of water on the surface of the sample material was measured by a static drop method. The measurement for each sample was repeated three times. The morphologies of the nano-TiO_2_ and the TiO_2_-KH570 were observed by TEM (H-800 model, Hitachi, Tokyo, Japan). The nano-TiO_2_ and the TiO_2_-KH570 powders were dispersed in deionized water. Each sample was ultrasonicated at 80 W for 1–2 min, dropped on a copper net, and air dried prior to the measurements. The morphology of PEEK was observed by a SEM (S-3400 model, Hitachi, Tokyo, Japan). A small amount of PEEK powder was evenly spread on the conductive adhesive and coated with gold.

### 2.4. Preparation of PMMA -TiO_2_-KH570 Composite Resin

The prepared TiO_2_-KH570 (1 or 2 wt. %) and PEEK (0, 1, 2, or 3 wt. %) NPs were dispersed in ethanol. The PMMA photosensitive resin (NDBAPI01000) and these NPs were mixed together according to Table 1. The mixture was then mechanically stirred at 500 rpm/min for 1 h, followed by ultrasonication at 40 W for 1 h. The mixture was then placed into a vacuum oven to remove air bubbles and residue ethanol at 50 °C. The prepared composite resins (Group-1 to -6, as listed in Table 1) were used to print specimens by the Digital Light Projection (DLP) Photocuring 3D printing system (Envision Tech, Gladbeck, Germany).

### 2.5. Mechanical Evaluation of PMMA Composite Resins (Group-1 to -5)

The mechanical properties of the PMMA composite resin, including flexural strength, flexural modulus, rupture work, and maximum stress intensity factor, were determined by a universal testing machine (UTM-5KN model, Shenzhen Sansi, Shenzhen, China) according to the standard of YY 0270.1-2011/ISO 20795-1:2008 Dentistry-Base polymers-Part 1: Denture base polymers [41]. Briefly, the specimen bars (64 mm × 10 mm × 3.3 mm) were printed by the DLP photocuring 3D printing system (Envision Tech, Gladbeck, Germany). Each sample was stored in water for 50 ± 2 h at 37 ± 1 °C prior to the three-point bending test. The loading speed of the bending test was changed by a constant 5 ± 1 mm/min until the sample broke. Before the fracture toughness test, each group sample was stored in water at 37 ± 1 °C for 7 d ± 2 h and then placed in water at 23 ± 1 °C for 60 ± 15 min before the test. During the test, the load head was gradually increased from zero at a constant displacement of 1 ± 0.2 mm/min until the maximum pressure was applied and the gap almost reached the opposite side of the test strip. When the load was reduced to 5% of the maximum load, the test was finished. Six specimens were prepared for each PMMA composite resin.

Fracture surfaces of PMMA composite resins were observed by S-4800 SEM (S-3400 model, Hitachi, Tokyo, Japan). Each specimen was washed with ethanol to remove residues on the cross section and air dried, followed by Gold (Au) coating. The distribution of TiO_2_-KH570 in the PMMA matrix across the fracture section was investigated through Ti element mapping.

### 2.6. Antibacterial Assessment of PMMA Composite Resins (Group-1 to -5)

Antibacterial activities of each group sample were evaluated using pathogenic bacterial strains of *Staphylococcus aureus* (*S. aureus,* Gram-positive bacteria) and *Escherichia coli* (*E. coli*, Gram-negative bacteria), which were kindly provided by Jinan University, China. The bacterial concentration was adjusted to 10^7^ CFU/mL by the addition of Luria-Bertani (LB) liquid medium. Each sample was added to the bacterial medium at a ratio of 1.25 cm^2^/mL of bacterial surface area and incubated in a CO_2_ incubator at 37 °C for 24 h. Then, 20 μL of the bacterial solution was taken and uniformly seeded on the LB agar plate, and the growth of the colony was observed after incubation at 37 °C for 24 h. Another 100 μL of the bacterial solution after the first 24 h incubation was taken to measure the optical density (OD) value of the bacterial solution at a wavelength of 450 nm, as previously described [42].

### 2.7. Cytotoxicity Assay of PMMA Composite Resins (Group-1 to -5)

CCK-8 assay was used to evaluate the cytotoxicity of PMMA composites. The sterilized samples were immersed in 10 mL Dulbecco’s modified Eagle’s medium (DMEM, Sigma, St. Louis, MO, USA) for 24 h according to ISO 10993-12:200. The extract was then added to the cell culture, and the surface to volume ratio of the sample to the medium was 1.25 cm^2^/mL. L929 fibroblasts (provided by Jinan University, Guangzhou, China) were cultured in DMEM supplemented with 10% fetal bovine serum (FBS, Life technologies), 100 IU/mL penicillin (Sigma), and 100 mg/mL streptomycin (Sigma) at 37 °C with 5% CO_2_ and 100% relative humidity. A seeding density of 1 × 10^4^ cell was co-cultured with the extracts of each sample in a 96-well plate at 37 °C with 5% CO_2_ and 100% relative humidity. The medium was replaced every two days. At days 1, 3, 5, and 7, 20 μL of CCK-8 solution was added into each well and incubated at 37 °C for 4 h. The OD values of samples were recorded at a wavelength of 450 nm using a microreader (Bio-Rad 680, Bio-Rad Laboratories, Hercules, CA, USA). Cells grown in medium without extracts were used as the negative control.

### 2.8. Blood Compatibility Assessment of PMMA Composite Resins (Group-1 to -5)

#### 2.8.1. Hemolysis of PMMA Composite Resins (Group-1 to -5)

Each PEEK composite sample of 5 g was mixed with 0.9% sodium chloride injection of 10 mL at room temperature for 48 h. The blood used in this assessment was provided by healthy volunteers. Red blood cells (RBC) of 800 μL were suspended in 4.2 mL of phosphate buffered saline (PBS) to prepare a 16% *v*/*v* red blood cell suspension. A sample solution of 1 mL was transferred into a centrifuge tube, and 16% red blood cell suspension of 50 μL was added into the same tube. The solution was mixed well and incubated at 37 °C for 4 h, then vortexed evenly and centrifuged at 1000× *g* for 5 min to sediment the red blood cell. A supernatant (200 μL) was pipetted into a 96-well plate, and the absorbance values of the released hemoglobin (Hb) in the supernatant at 540 nm was measured by a microplate reader (Multiskan MK3, Thermo Scientific, Waltham, MA, USA). A positive control was the RBC suspension (16% in PBS, *v*/*v*) of 50 μL in 1 mL of water, while a negative control was the RBC suspension (16% in PBS, *v*/*v*) of 50 μL in 1 mL of PBS. Each sample group was performed in parallel three times. Hemolysis was calculated according to the following formula:

(1) $$\text{Hemolysis}\;\left(\%\right)=\frac{A_{S}-A_{n}}{A_{p}-A_{n}}\times 100\%$$

where A_s_, A_n_, and A_p_ are the absorbance of the sample, the negative control, and the positive control, respectively.

#### 2.8.2. Activated Partial Thromboplastin Time (APTT) and Prothrombin Time (PT)

The anticoagulant whole blood was centrifuged at 1000× *g* for 15 min, and the supernatant was collected to obtain platelet-poor plasma. Then, 180 μL of supernatant was mixed with 20 μL of sample material solution (PEEK composite resin in 0.9% NaCl, as previously described). After adding the corresponding reagents, the activated partial thromboplastin time (APTT) and the prothrombin time (PT) of the samples were measured with an automatic coagulation analyzer (STAR Evolution, Diagnostica Stago, Assiernes, Asnières-sur-Seine, France). PBS was used as a control group in the clotting analysis. Each sample group was performed in parallel three times. The reagents and the consumables used in the coagulation analyzer were provided by the First Affiliated Hospital of Jinan University.

### 2.9. 3D Printing Trials

To test the compatibility of the newly prepared PMMA-TiO_2_-KH570-PEEK composite resin, denture models were printed using a Vida model of a digital light projection (DLP) photocuring 3D printing system (Envision Tech, Gladbeck, Germany) as previous described [42]. Briefly, a functional model of maxillary edentulou arch was scanned using a DL-100 intraoral scanner (Guangdong Langcheng Medical Device Technology Co. Ltd., Guangdong, China). The 3D software (3Shape, 2017) was used to design a model of a denture with the scanned data, which were converted into a stereolithography (STL) file. The 3D printing parameters were set to a thickness of 100 μm in the Z-axis layer, a single layer exposure time of 4.4 s, and an ultraviolet light intensity of 1700 μm/cm^2^. The ultraviolet light-curing composite resin was projected by the light machine when the printer was in operation. After each layer was cured, the printing platform was moved upward with the cured product, while the uncured liquid composite resin flowed back to the bottom. The printing platform was then lowered to a position 100 μm from the groove for the next layer of printing. The process was repeated until the dental base was complete. A final denture base model was obtained after the post-treatment procedure. The denture base model was cleaned with 95% ethanol solution and dried, then its support was removed and it was polished and cured using a photocuring box.

## 3. Results

### 3.1. Characterizations of TiO_2_, TiO_2_ -KH570 and PEEK

**Figure 1:**
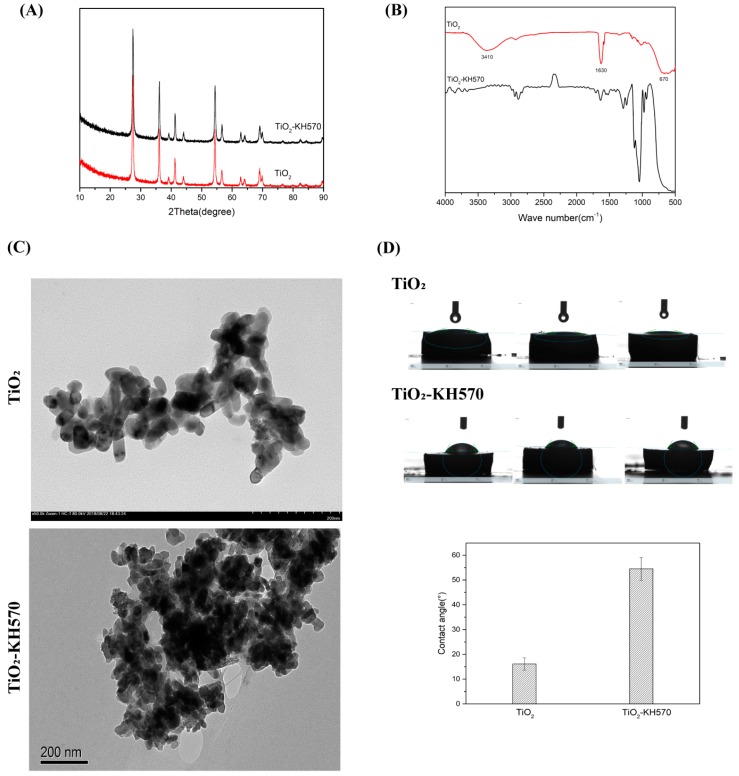
Characterizations of prepared NPs of TiO_2_ and TiO_2_ –KH570 by the use of XRD (**A**), FTIR (**B**), TEM (**C**), and contact angle test (**D**).

TiO_2_ NPs were prepared by hydrothermal synthesis and then modified by silane coupling agent to obtain TiO_2_-KH570. The X-ray diffraction patterns of TiO_2_ and TiO_2_-KH570 samples are shown in Figure 1A. The main crystallization peaks of TiO_2_ and TiO_2_-KH570 samples were similar to each other, which were at 27.45°, 36.08°, 41.22°, 54.32°, 56.64°, and 69.01°, corresponding to (1 1 0), (1 0 1), (1 1 1), (2 1 1), (2 2 0), and (30 1 1) for the crystal planes of rutile TiO_2_, respectively. This result suggested the crystallization peaks of the prepared TiO_2_ and TiO_2_-KH570 were in good agreement with the standard spectrum of TiO_2_ [43,44]. As shown in Figure 1B, the FTIR spectrum of nano-TiO_2_ showed a strong characteristic absorption peak of -OH at 3410 cm^−1^ and 1630 cm^−1^, confirming that the surface of the prepared nano-TiO_2_ was hydrophilic, as it was rich in hydroxyl groups. The absorption peaks at 800–500 cm^−1^ were attributed to the bonding of Ti-O-Ti [37]. After being treated with KH570, the stretching vibration peak of Si-O-Ti appeared near 1100 cm^−1^, indicating that KH570 was successfully grafted onto the surface of TiO_2_. TEM images (Figure 1C) showed that the synthesized nano-TiO_2_ was a standard nano-particle, which was an approximately elliptical particle with a major axis length of about 40 nm and a minor axis length of about 15–20 nm. After being modified with KH570, the particle size and the morphology of TiO_2_-KH570 were similar those of TiO_2_, but it seemed to be easy to aggregate together, probably due to its low solubility in water. The contact angle analyses of TiO_2_ and TiO_2_-KH570 are shown in Figure 1D. The contact angles of nano-TiO_2_ and TiO_2_-KH57 with water were about 18° and 55°, respectively, suggesting that the hydrophilicity of TiO_2_-KH57 was remarkably lower than that of TiO_2_.

**Figure 2:**
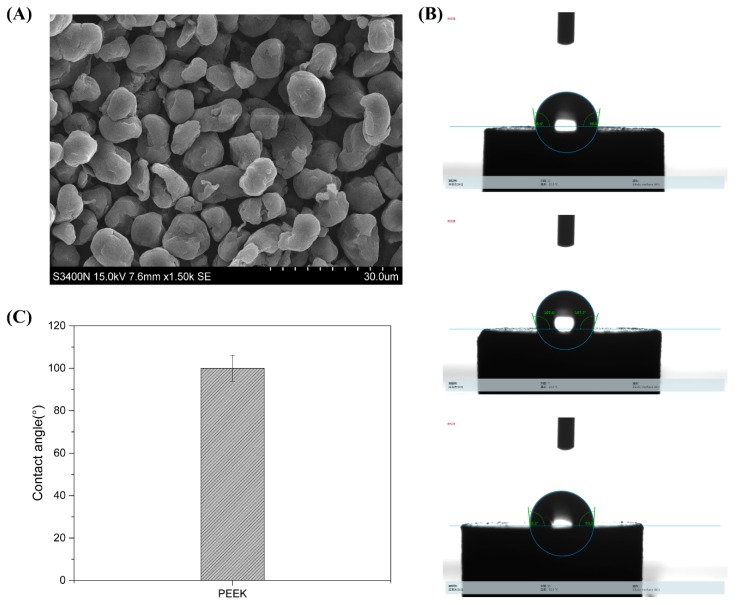
Characterizations of PEEK micro-particles using SEM (**A**) and contact angle test (**B**,**C**).

As shown in Figure 2A, PEEK had an irregular, small spherical shape and a particle diameter of about 10 μm, which was a standard micro-particle. The contact angle of PEEK was about 95°, suggesting the nano-material was hydrophobic (Figure 2B,C).

### 3.2. Mechanical Evaluation of Different PMMA Composite Resins

**Figure 3:**
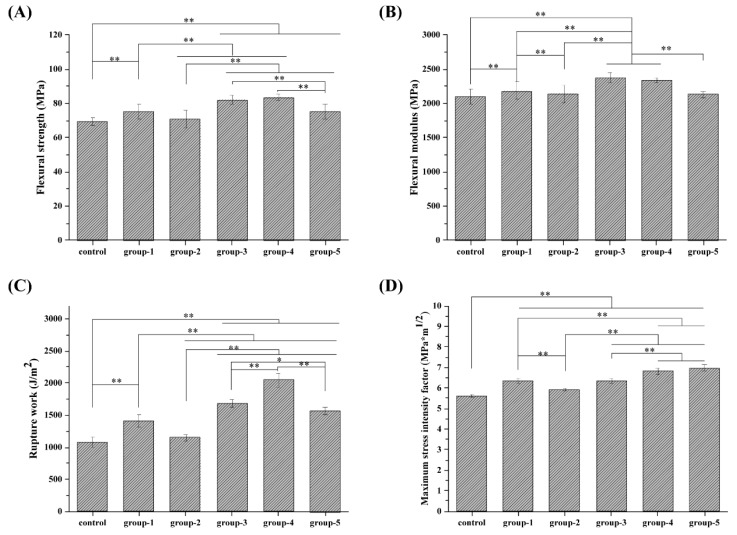
Mechanical properties of PMMA composites resins: (**A**) flexural strength; (**B**) flexural modulus; (**C**) rupture work; and (**D**) maximum stress intensity factor.

The average bending strength and the flexural modulus of PMMA resin (the control group of TiO_2_ -0%-PEEK-0%) were 69.2 ± 2.34 MPa and 2100.05 ± 114.28 MPa, respectively, as shown in Figure 3A,B. With an addition of 1% of TiO_2_, the flexural strength and the flexural modulus of the composite resin (Group-1 of TiO_2_-1%-PEEK-0%) were improved to 75.26 ± 4.25 MPa and 2181.78 ± 128.95 MPa, respectively, which were increased by 8.6% and 3.9%, respectively, compared to the control group. For Group-2 with 2% of TiO_2_, the flexural strength and the flexural modulus were 70.75 ± 5.24 MPa and 2143.58 ± 127.15 MPa, respectively, which were not significantly different from the control group. Group-3 (TiO_2_-1%-PEEK-1%) and Group-4 (TiO_2_-1%-PEEK-2%) showed the highest average bending strength and flexural modulus, at least 10% higher than the other groups. Though the average bending strength and the flexural modulus of Group-5 (TiO_2_-1%-PEEK-3%) were slightly higher than those of the control group, they were still significantly lower than those of Group-3 and Group-4.

In terms of rupture work, as shown in Figure 3C, Group-2 was higher than that of the control group and Group-3, indicating that the addition of 1% TiO_2_ was an effective amount to enhance the mechanical properties of the composite resins. In addition, the impact strengths of Groups-3, -4, and -5 were significantly higher than that of Group-1, indicating that the addition of PEEK could significantly improve the mechanical properties of the composite resin. For example, the rupture work of Group-4 was almost 2-fold and 1.6-fold higher that of the control group and Group-1. The results showed that the maximum stress intensity factor (Figure 3D) pattern was similar to that of the impact strength results. The maximum stress intensity factors of Groups-1, -3, -4, and -5 were all higher than that of the control group and Group 2.

Combined with the experimental results, Group-4 showed the most optimized mechanical properties. It had a better average bending strength and impact strength than other groups, only the flexural modulus was slightly lower than Group-3, and the maximum stress intensity factor (6.84 ± 0.15 MPa) was slightly lower than Group-5 (6.96 ± 0.15 MPa).

### 3.3. Fracture Surface Analysis of Different PMMA Composite Resins

**Figure 4:**
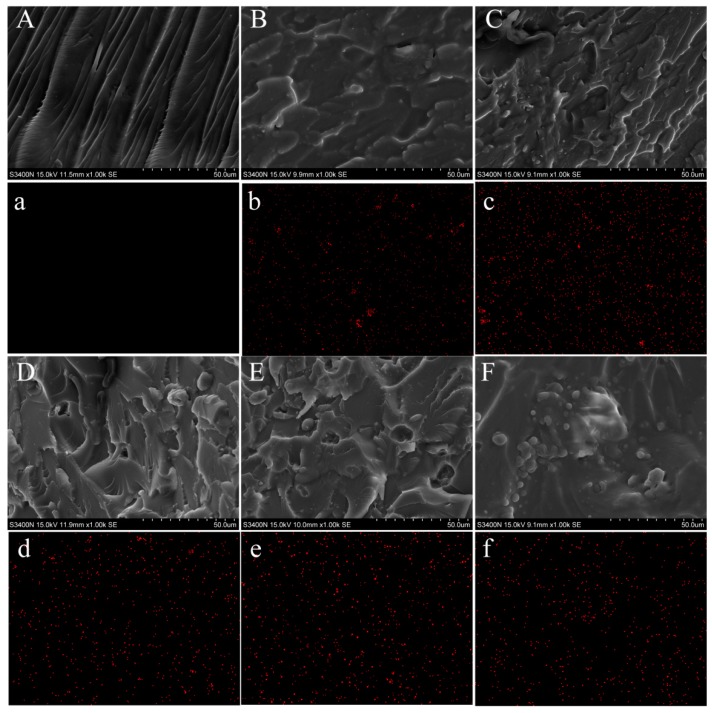
SEM images (**A**–**F**) and titanium element mapping images (**a**–**f**) for the fractured surfaces of PMMA composite resins after flexural testing: Control (**A**-**a**); Group 1 (**B**-**b**); Group 2 (**C**-**c**); Group 3(**D**-**d**); Group 4 (**E**-**e**); and Group 5(**F**-**f**).

Figure 4 shows the SEM images of the fractured surfaces of composite resins, and the distribution of TiO_2_-KH570 in the resins could be observed by the element distribution of Ti. The cross-section of the control group (TiO_2_-0%-PEEK-0%) showed classic characteristics of brittle fracture with some sharp edge cracks. By introducing the modified nano-TiO_2_ into the composite resin, the cross-sections of the other groups (Figure 4B–F) were rougher than the control group. Some small embedded silver spots were found in the PMMA composite resins (Figure 4B–F), which were caused by the addition of TiO_2_. Figure 4D–F shows that the presence of PEEK became more apparent due to the increase of PEEK in the PMMA composite resins. As shown in Figure 2, PEEK were small spheres with a size about 10 μm. For Group-3 (TiO_2_-1%-PEEK-1%), PEEK were scattered and embedded in the composite resin (Figure 4D). For Group-4 (TiO_2_-1%-PEEK-2%), the amount of the round particles (i.e., PEEK) was significantly increased in the composite resin, while the PEEK were still able to disperse uniformly in the resin. As shown in Figure 4F, there were some PEEK particle aggregations found in the cross-section of Group-5 (TiO_2_-1%-PEEK-3%). The mapping of Ti element was used to further confirm the distribution of the modified nano-TiO_2_ in the composite resin (Figure 4a–f). The Ti element signal of Group-3 (Figure 4c) was slightly stronger than that of the other groups. This was due to fact that the content of TiO_2_-KH570 in Group-2 was 2 wt. %, higher than that of the other groups (with nil or 1% TiO_2_). In addition, all the samples showed reasonable signal intensities of Ti element rather than being too rich, indicating that the TiO_2_-KH570 were uniformly distributed in the composite resins.

### 3.4. Antibacterial Activity of Different PMMA Composite Resins

**Figure 5:**
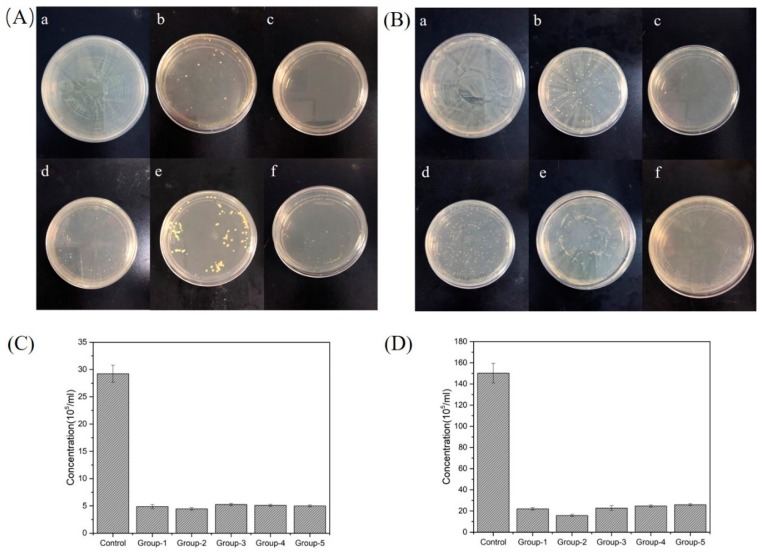
Antibacterial activity of different PMMA composite resins containing TiO_2_ or/and PEEK. Agar plates displaying the samples with: (**A**) *Staphyloccocus aureus*; (**B**) *Escherichia coli*. The change of bacterial concentration by co-culturing the samples with: (**C**) *Staphyloccocus aureus*; (**D**) *Escherichia coli*.

The antibacterial effects of PMMA composite resins were carried out by co-cultivating a sample with a bacterial strain on agar plates, in which *Staphylococcus aureu* was cultured on tryptic soy agar (TSA), and *Escherichia coli* was cultured on LB agar medium. The plates were then cultured at 37 °C for 24 h. The colony growth of *S. aureu* and *E. coli* is shown in Figure 5A,B, respectively. The control group showed no antibacterial properties against either *E. coli* or *S. aureus*, as the colonies were covered with the entire agar plate. Group-2 (TiO_2_–2%-PEEK–0%) showed the strongest antibacterial properties compared to the other samples, as no colonies were formed on the plate. Groups-1, -3, -4, and -5 also showed good antibacterial effects against the experimental strains, as few colonies were observed on the plates. The difference between these groups was not distinguished, indicating that the composite resins containing 1 wt. % of TiO_2_ had good antibacterial effects on *E. coli* and *S. aureus*.

The samples were also incubated with experimental strains for 24 h, and the OD values of the bacteria solutions were determined, as shown in Figure 5C,D. The results showed that, compared to the control group, the concentration for the other groups (i.e., Groups-1, -2, -3, -4, and -5) had all sharply decreased to less than 1/5 and 1/7.5 in the culture of *S. aureu* and *E. coli,* respectively. In addition, similar to the results of bacteria-agar plate experiment, Group-2 (TiO_2_-2%-PEEK-0%) showed the most excellent antibacterial properties compared to the other groups, especially for the case of co-culturing with *S. aureu.*

### 3.5. Cytotoxicity of PMMA Composite Resins

**Figure 6:**
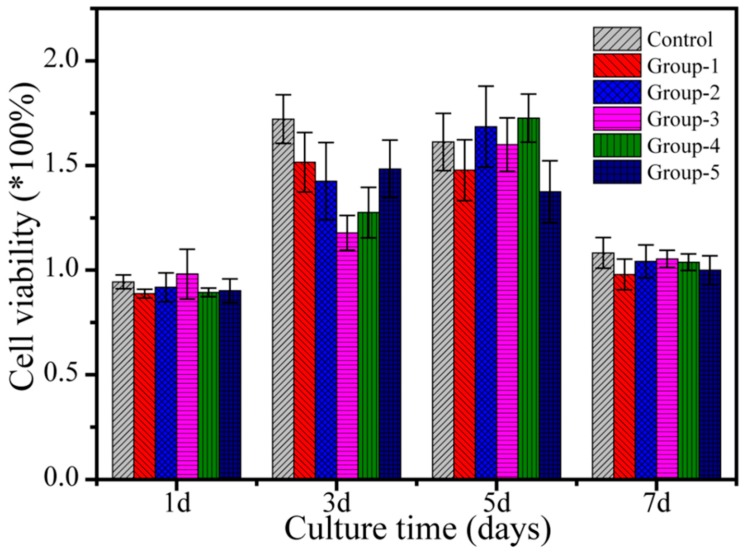
Cytotoxicity assay by culturing L929 fibroblasts in extracts from different PMMA composite resins (Control and Group-1 to -5) according to ISO 10993-12:200. The cell proliferation rates were normalized to the optical density (OD) values of the negative control.

A cytotoxicity test was performed according to ISO 10993-12:200 standards. Extracts of each sample were prepared and incubated with L929 fibroblast culture, and L929 fibroblast culture alone was used as a negative control. The cell growth rate of each sample was normalized to that of the negative control, as shown in Figure 6. On the first day, the survival rate of L929 fibroblasts of each sample was slightly lower than that of the negative control group. On days 3 and 5, L929 fibroblasts showed a significant proliferative tendency in the extracts of each sample group compared to the negative control. On day 7, the cell viability of each sample was close to that of the negative control group and was lower than that on day 5. Cytotoxicity results showed that all the prepared PMMA composite resins had good cytocompatibility and may have had some effect on promoting cell activities.

### 3.6. Effects of Different PMMA Composite Resins on Blood Compatibility

**Figure 7:**
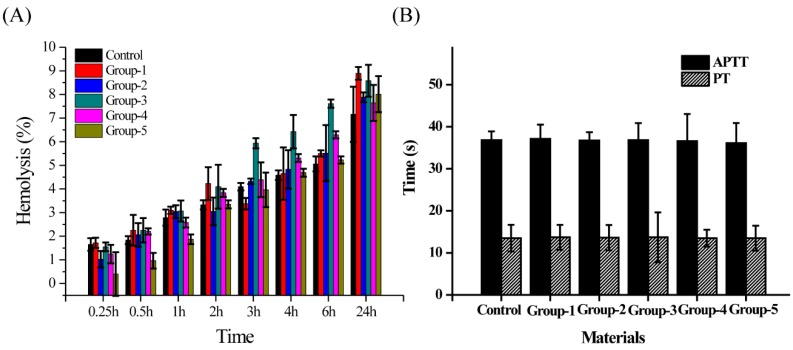
Effect of different PMMA composite resins on (**A**) Hemolysis; (**B**) activated partial thromboplastin time (APTT) and prothrombin time (PT).

This study aimed to develop practical PMMA composite resins for clinical use, which would directly contact human blood. If the composite materials do not meet the requirements of blood compatibility performances, it not only affects their clinical application in the oral field but also poses potential risks to the health of patients. Therefore, the blood compatibility tests were performed on different PMMA composite resins, including hemolysis APTT and PT, as shown in Figure 7. The results showed that all the hemolysis rates of these six materials were less than 5% at the first 4 h. Later, the hemolysis rates of all samples slowly increased over time. At 24 h, the hemolysis rates of the six materials ranged from 7 to 9%. APTT and PT are routine test parameters for plasma coagulation. In Figure 7B, the APPT of the six materials was about 36.5 s, and the PT was about 13.5 s, which was within the normal range. Therefore, it can be concluded that the six PMMA composite resins had less damage to red blood cells under the experimental conditions and showed good blood compatibility.

### 3.7. 3D Printing Outcomes

**Figure 8:**
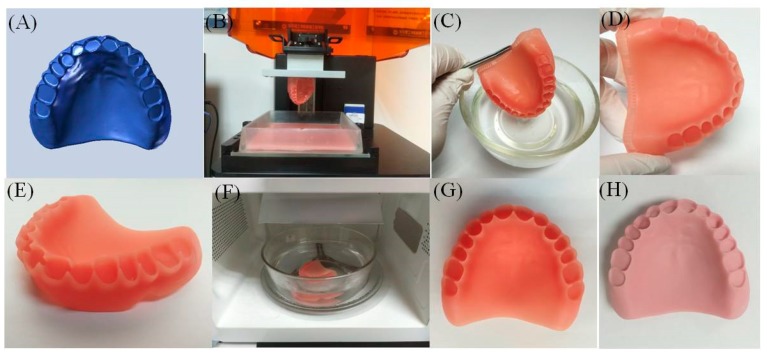
Printing process and product effect of denture base using PMMA resin and PMMA composite resin containing TiO_2_–1%-PEEK-1%. (**A**) Designing a base model; (**B**) printing and removing the base; (**C**) cleaning the base; (**D**) drying the base; (**E**) removing support and sanding; (**F**) secondary curing base; (**G**) 3D printed denture base using PMMA resin; and (**H**) PMMA composite resin containing TiO_2_-1%-PEEK-1%.

The denture base models of PMMA pure resin (control group) and PMMA (TiO_2_-1%-PEEK-1%) composite resin (Group-4) were prepared according to the protocol (for details, refer to the Materials and Method section). The production process and the printing effect are shown in Figure 8. A model made of pure PMMA resin was orange pink (Figure 8G), while the other model made of PMMA (TiO_2_-1%-PEEK-1%) composite resin was light pink (Figure 8H). This was due to the fact that both TiO_2_ and PEEK were white NPs. The addition of a small amount of these NPs resulted in a slightly whiter appearance of the denture base. In addition, both models showed a smooth surface with no visible layered pattern, indicating that the prepared PMMA (TiO_2_-1%-PEEK-1%) composite resin can be used for 3D precision printing.

## 4. Discussion

The materials of the dental denture base should have excellent properties, such as stable chemical properties, good physical and mechanical properties, easy to polish, non-toxic, and antibacterial properties. In this study, we introduced modified nano-TiO_2_ to enhance the antimicrobial properties of the PMMA resin base. The nano-TiO_2_ was prepared via a hydrothermal method and then modified by silane coupling agent KH570. The spectra of FTIR and XRD indicated that TiO_2_ and TiO_2_-KH570 NPs were successfully prepared, and the TEM images showed that the prepared TiO_2_ and TiO_2_-KH570 NPs both had an elliptical shape with a long axis and a short axis length of 40 nm and 15–20 nm, respectively. In addition, the TiO_2_-KH570 NPs were more aggregated than TiO_2_ NPs, which was due to the fact that the modification of KH570 reduced the hydrophilicity of the TiO_2_ NPs. This result was consistent with the water contact angle analysis (Figure 1D), which showed that the contact angles of nano-TiO_2_ and TiO_2_-KH570 with water were about 18° and 55°, respectively. The lower the contact angle of the material with water is, the better the hydrophilicity of the material is. This result indicated that the prepared TiO_2_-KH570 NPs were more hydrophobic than TiO_2_ NPs, which contributed to a better dispersion of TiO_2_-KH570 NPs in the complex resin. PEEK is an engineering plastic with excellent performance and properties such as good mechanical strength, light weight, non-toxic, strong corrosion resistance, etc., showing great potential in oral and orthopedic applications [16,17,18]. Herein, we introduced PEEK to enhance the general mechanical performance of the PMMA resin base. Figure 2 shows that the prepared PEEK particles were irregular, small spherical shapes with particle diameters of about 10 μm, and the water contact angle was more than 90°.

The combination of TiO_2_ and PEEK was added to the PMMA resin, which greatly improved the average bending strength and the flexural modulus (Figure 3). The addition of nano-TiO_2_ increased the flexural strength and the flexural modulus of the resin compared to that of PMMA resin (control group), as shown in Figure 3A,B. However, when the TiO_2_ content was 2 wt. % (Group-2), the flexural strength and the flexural modulus of the composite resin were not significantly different from the control group. During the printing process, we also found that Group-2 encountered some printing problems, such as over-solidification and shedding, which could have caused the 3D printing process to fail. The possible reason for this was that the nano-material was easy to aggregate, and the defect formed in the PMMA resin matrix might have led to the degradation of the composite resin. In addition, the strong ultraviolet absorption effects of TiO_2_ NPs might have hindered the light curing process of PMMA. If the TiO_2_ NPs content exceeded a critical value in the resin matrix, it may have over-absorbed the ultraviolet light, resulting in defects such as voids and incomplete curing in the resin matrix. This might have led to a decrease in the mechanical performance of the composite resin and even failure to complete the curing process. Therefore, mechanical evaluation indicated that 1 wt. % of nano-TiO_2_ had an enhanced effect on the mechanical properties of PMMA composite resin, not only in terms of bending strength and flexural modulus, but also in the impact strength and the maximum stress intensity factor of the composite resins, as shown in Figure 3C,D. To further improve the mechanical properties of PMMA composite resin, PEEK was used as a micro-filler combined with nano-TiO_2_ to reduce the aggregation of the fillers in the resin matrix. The results showed that the addition of PEEK could effectively improve the mechanical properties of the resin samples, especially the impact strength of the resin. As shown in Figure 3C, the impact strengths of Group-3, Group-4, and Group-5 were significantly higher than that of the control group, with Group-4 increasing by up to 90%. This might have been due to the fact that, when the material was subjected to severe impacts, the polymer matrix around the filler caused yield, absorbing large amounts of deformation work. This process would hinder the crack propagation in the composite resin and prevent the destructive cracking from absorbing energy. Since the deformation rate of the filler under stress is small, the interface between the matrix and the filler is partially debonded to generate voids. These voids may cause crack passivation, hindering the cracks from expanding into destructive cracks and thereby producing toughening effects.

The SEM and the mapping analysis of the fractured cross-section of the composite resins further explored the effects of these nano-fillers on the mechanical properties of the PMMA composite resin. Group-2 (TiO_2_–2%-PEEK–0%) had weaker mechanical properties than the other composite resin groups. In Figure 3C, the Ti element signal of Group-2 showed the strongest local signal, indicating that TiO_2_-KH570 might aggregate, affecting its mechanical properties. For the addition of PEEK, as the PEEK content increased, the particles that appeared on the fractured surface of the resin matrix increased significantly. A large amount of aggregates was clearly shown in the cross-section when the PEEK content reached 3 wt. % (Figure 4f, Group-5). Though the interface between the nano-filler and the resin was well bonded, the decreased mechanical properties caused by the internal defects of filler aggregation were still unavoidable. The partial nano-fillers aggregated in the resin matrix, which might have become defects after the composite resin was cured. The higher the defect rate is, the lower the mechanical properties of the composite resin are.

The cytocompatibility of the material is a critical factor that determines whether the implant material can be used in the human body. If a small amount of unpolymerized residue monomers or other components in the resin may precipitate out of the resin, there may be a potential risk to the activity of oral tissues. The results of cytocompatibility (Figure 6) showed that the survival rate of L929 fibroblasts on the first day of all groups was slightly lower than the negative control group. This was due to the slower cell proliferation on day 1, thus the activity of the samples was relatively low. When the cells were cultured until day 3, L929 fibroblasts were able to proliferate in the extracts of all group samples, which was comparable with the negative control. When the cells were cultured until the day 7, the cell concentration did not continue to grow and approached the maximum allowable amount for the environment, making it subject to external conditions such as space and nutrients. Therefore, the cell concentrations of all group samples on day 7 were lower than those on the first day, and the cell concentrations on day 5 were close to the negative control group. The experimental results showed that PMMA composite resins have good cytocompatibility and may exert some effect on promoting cell activities.

The PMMA composite resins, as promising implants in the oral cavity, are inevitably in contact with blood. If the composite material and the blood are incompatible, there will be some side effects, harming the human body. Therefore, we performed hemolysis, APTT, and PT tests on different PMMA composite resins. Several studies reported that the material may have a serious impact on red blood cells when the hemolysis rate of a material is higher than 20% [45,46]. APTT is a sensitive test for screening whether the endogenous coagulation system is normal. The simultaneous detection with PT is the main screening test combination of the second stage hemostasis. These two methods are important indicators of evaluations on biomaterials blood compatibility, and the normal ranges of APTT and PT are 27–40 s and 11–14 s, respectively. Figure 7 shows that the hemolysis rates of these six samples were between 7% and 9%, and the APPT and the PT of these six samples were in the normal range. Therefore, the prepared PMMA composite resins cause little damage to red blood cells and do not cause an activated coagulation pathway or a coagulation exogenous pathway, showing good blood compatibility.

Combining the results of the mechanical properties, the cytotoxicity, the antibacterial, and the hemolysis experiments, PMMA (Group-4, TiO_2_–1%-PEEK-1%) composite resin was proposed as the most optimized printing resin in this study. In the DLP light curing 3D printing trial, the denture model printed by PMMA (TiO_2_–1%-PEEK-1%) composite resin showed a smooth surface and a precise resolution. Further research will focus on optimizing the microwave light–heat conditions on the light-curing resins, and the finite element static analysis will be used to systematically evaluate the stress of the 3D printed denture bases in the oral cavity.

## 5. Conclusions

The reinforced PMMA composite resins with different ratios of TiO_2_ and PEEK were successfully prepared in this study. The addition of TiO_2_ in the composite resin showed excellent antibacterial properties compared to pure PMMA resin. The addition of TiO_2_ (1 wt. %) and PEEK (1–3 wt. %) in the composite resin enhanced the mechanical strength in a synergetic way, which was better than the addition of TiO_2_ alone. The reinforced composite resins of PMMA (TiO_2_-1%-PEEK-1%) showed great potential as functional dental restoration material due to their excellent mechanical strength, high antibacterial activity, and low cytotoxic effects. In addition, the reinforced composite resins prepared in this study have a simple preparation process and consequently high operability and low production cost, thus showing great potential in dental applications.

## Figures and Tables

**Table 1:** Composition of polymethyl methacrylate (PMMA) composite resin for different groups.

PMMA Composite Resin	Resin Base (96–100 wt. %)	Nano-Filler (0–4 wt. %)	
	PMMA (g)	TiO_2_ (g)	PEEK (g) *
Control	100	0	0
Group-1	99	1	0
Group-2	98	2	0
Group-3	98	1	1
Group-4	97	1	2
Group-5	96	1	3

* PEEK = polyetheretherketone.
